# Awareness and Knowledge about Occupational Therapy in Jordan

**DOI:** 10.1155/2018/2493584

**Published:** 2018-05-21

**Authors:** Wesam Barakat Darawsheh

**Affiliations:** Department of Occupational Therapy, School of Rehabilitation Sciences, The University of Jordan, Amman, Jordan

## Abstract

Knowledge and awareness about occupational therapy (OT) are essential for the delivery of quality care to all clients and for occupational therapists' (OTRs) job satisfaction. OT has been a poorly understood profession in Jordan. The current study reports on the assessment of Jordanians' awareness and knowledge of occupational therapy. Convenience sampling was used. There were 829 participants (474 males, 355 females), with mean age of 32 ± 11.6 yrs. They were recruited from the three main geographical areas of Jordan (northern, central, and southern) and from all educational levels. The sample included 222 (26.8%) healthcare personnel, 146 (17.6%) clients, and 461 (55.6%) lay persons. Participants completed questionnaires, and the results revealed that 48% of the sample had poor or no knowledge about OT, while 28.3% were unaware of it. Also, OT was commonly (50%) perceived to be exclusively targeting people with disabilities (PWDs) and neurological and physical conditions (58% and 53%, resp.) in addition to exclusively providing services for the rehabilitation of the upper extremity (48%). Common misconceptions associated with OT were that OTRs prescribe medication (43%) and OTRs are physiotherapists (44%). These preliminary findings suggest that efforts need to be directed by OTRs, the Jordanian Society of Occupational Therapy (JSOT), and the Ministry of Health to preserve the OT identity and value and promote knowledge about OT in the public and among members of interdisciplinary teams. More interprofessional learning needs to be incorporated within the curricula and placements of all healthcare personnels.

## 1. Introduction

Occupational therapy (OT) is one of the components of a multidisciplinary team approach that focuses on enabling participation in meaningful occupations as an essential element of healthcare [1–3]. Generally, the conceptualization of OT has been vague among the public [4] and even among healthcare professionals [5]. This becomes more evident in some countries with low and lower middle-income economies, such as Jordan and Nigeria [6, 7], and in some clinical settings such as mental health and school-based settings [4, 8].

Knowledge about OT among healthcare professionals is essential to guarantee the delivery of comprehensive, holistic, and quality care services [3, 6]. Healthcare professionals need to understand other team members' roles so that appropriate referrals can be made and to prevent role confusion [9]. Clients can sometimes be deprived of OT services because healthcare professionals lack knowledge about the services provided by registered occupational therapists (OTRs) [6, 9]. In addition, other healthcare professionals' knowledge about OT affects the status of the profession and the level of job satisfaction among OTRs [10].

Carrier et al. [11] and Kristensen et al. [12] found that decision making about suitable methods for implementing evidence-based practices is affected by the cultural knowledge of the therapists and the way culture influences healthcare practices. Shafaroodi et al. [13] found that public knowledge about OT affects the process of clinical reasoning among OTRs. They found that managers have erroneous expectations concerning the role of OTRs which, in return, affects the process of treatment planning and decreases the quality of outcomes of OT intervention [13]. The lack of knowledge of healthcare professionals about OT reduces the opportunities of engaging in mutual planning and reasoning with OTRs which reflects negatively on the quality of care [13]. The level of knowledge of clients about OT affects their expectations and acceptance of OT services [13]. OTRs often find themselves iteratively altering their treatment plans to enable clients to overcome issues related to denial, over or under expectations, or lack of acceptance of OT services. Thus, OTRs have to face issues related to confidence where they continuously question their abilities and skills to meet the clients' demands [13].

Vincent et al. [14] explored the perceptions of four primary school teachers in South Australia concerning OT. OT was perceived to be useful and needed. However, teachers expressed a need to develop further channels of collaboration and interaction between educators and OTRs to maximize educational outcomes. Benson et al. [15] surveyed 47 public school teachers (37 special education and 10 regular education teachers) concerning OT in Pennsylvania. Teachers taught children from preschool age to 12th grade and had experience working with OTRs before. They expressed the valuable contribution of OT in the educational setting, but that it needed to be further supported by the educational system as more collaboration between educators and OTRs was required. In these studies, participants acknowledged the value of OT in multidisciplinary teams, but they could not identify or define the role of OTRs. Participants perceived OT as an extra pair of hands for care provision when there was an excessive caseload, and the value of OT was not based on a genuine understanding of the uniqueness of the role and services provided.

Smith and Mackenzie [16] investigated the knowledge of nurses about OT. Participants expressed that they had to work with OTRs by guessing their role due to a lack of information, interprofessional communication, and collaboration. Olaoye et al. [6] investigated the knowledge and awareness of OT in 581 Nigerian undergraduate healthcare students (medicine, dentistry, nursing, and rehabilitation). They found that even though 80% of the students were aware of OT, 62.3% of them had poor to moderate actual knowledge about the OT role. Factors such as specialization and level of education were found to influence the level of knowledge and awareness about OT. Such studies did not consider public knowledge or the knowledge of healthcare professionals from all healthcare backgrounds such as para healthcare professionals (e.g., social work and counseling professionals and medical laboratory professionals) and associate healthcare professionals (e.g., medical and pharmaceutical technicians and nursing and midwifery associate professionals). Research to date indicates that further information is required about public and healthcare professionals' knowledge about OT and factors that may influence knowledge and awareness about OT in each unique cultural context.

OT in the Middle East was first introduced in Jordan in 1986 [17] and is therefore a relatively young profession especially when compared to other healthcare professions [17, 18]. Only two studies have been conducted to explore the knowledge about OT in Jordan. Abu Tariah et al. [18] investigated the knowledge of 556 healthcare professionals, who were nurses, physicians, and physiotherapists, about OT. AlHeresh and Nikopoulos [17] investigated the knowledge of 153 participants (physicians, nurses, and rehabilitation specialists) about OT. In both studies, the same hospitals located in the capital city of Amman were targeted. They were general hospitals offering OT services for clients from all age ranges and for physical and neurological conditions. Although the results of both studies reflected a limited knowledge about OT in Jordan, they could not be generalized to all geographical areas in Jordan, to the general public, or even to all categories of healthcare professions. It has been over five years since these investigations were conducted, and there is a need for current studies to determine any changes in the level of knowledge about OT. Therefore, the aim of the current investigation was to explore the level of knowledge and awareness about OT in the general public and among clients who had/were receiving OT services in all areas of Jordan. Another purpose was to investigate the level of awareness and knowledge of healthcare professionals from the various healthcare fields that make up the rehabilitation team.

## 2. Methods

### 2.1. Participants

Participants were adults (age ≥ 18 yrs) from the three areas of Jordan (northern, central, and southern). Participants from all educational levels and occupations according to the International Standard Classification of Occupations (ISOC) [19] were included in the study. Healthcare professionals and workers in the healthcare field were included. OTRs/OT assistants were excluded to meet the aims of this study, which was to investigate the knowledge and perception of members of the community, who did not study the field of OT, about this profession, whether they were lay people, clients, or from other healthcare professions. Clients who received occupational therapy services were included. Lay people who had never received OT services and who were not healthcare professionals nor working in the healthcare field were also included.

### 2.2. Procedures and Recruitment

Ethical approval was granted by the Board of the Occupational Therapy Department and the Board of the Rehabilitation Sciences Faculty at the University of Jordan. Participants were approached in all of the three areas in Jordan in public and private institutions and settings. The lay people were approached in crowded areas such as stores, offices, schools, malls, and universities. The principal researcher used her personal and professional networks in clinics, healthcare centers, and hospitals to connect with healthcare professionals, most of whom assisted in approaching clients and inviting them to participate in the study. All participants were invited to complete a questionnaire after the aims of the study had been explained and participants had been encouraged to ask questions.

### 2.3. Assessment Tool/Instrumentation

The questionnaire was specifically developed for this study based on prior questionnaires that had been previously constructed in similar and pertinent studies conducted in Jordan, such as Abu Tariah et al. [18] and AlHeresh and Nikopolous [17], and in other countries, such as Benson et al. [15] and Olaoye et al. [6]. A draft of the questionnaire was reviewed by a group of nine occupational therapists working in the field. Their comments, suggestions, and feedback were taken into account to finalize the questionnaire.

The survey comprised seven sections: (1) background information (age, gender, occupation educational level, and area of living), (2) sources of knowledge of OT, (3) awareness and general evaluation of self and public knowledge/awareness about OT, (4) general knowledge about the aims and services of OT, (5) knowledge about OT domains and places of service provision, (6) misconceptions about OT, and (7) means to promote knowledge and awareness about OT. Questions in sections 1, 2, 3, 7, and 8 included options from which participants could make a selection, and an option of “other” was listed in case none of the listed options met their answer. Sections 4, 5, and 6 were the main sections of the survey. They comprised 40 questions/statements against which the knowledge of OT was scored out of 80. Statements in sections 4 and 5 included actual facts about OT while statements in section 6 included statements that examined the common misconceptions about OT. Answers in sections 4, 5, and 6 were closed ended where participants were required to choose one answer from three options (Yes, I do not know, No) against each statement. Each answer was scored from 0 to 2 where Yes = 2, Do not know = 1, and No = 0, except for the misconception section (section 6) where answers were scored as Yes = 0, Do not know = 1, and No = 2. This method of scoring was followed so that the better the knowledge about OT, the higher the total score.

### 2.4. Data Analysis

Statistical analysis was performed using SPSS Version 22.0 (2016, IBM Corporation, New York). Descriptive statistical analysis was employed to report on the frequency of categorical variables. The comparisons of the scores in sections 4–6 between groups classified by age, gender, educational level, area (northern, central, and southern), and status (lay, client, or healthcare personnel (HP)) were performed by the multivariate analysis of variance MANOVA. The Tukey post hoc test was utilized to identify the subgroup comparisons that caused significant differences.

Educational level was subcategorized into five subcategories: primary and elementary school levels (Sch), high school (HS), associate degrees (Asso), bachelor degrees (Bch), and master and doctoral degrees (MA+). This classification follows the common classification of academic degrees [20]. Participant age ranges were subcategorized into five subcategories as per the most common standardized method of classification used in surveys: 18–24 yrs (A), 25–34 yrs (B), 35–44 yrs (C), 45–54 yrs (D), and 55+ yrs (E) [21, 22].

The reliability assessment of the developed survey in this study was implemented by calculating the value of the internal consistency reliability coefficient (Cronbach's alpha reliability coefficient). A conventional benchmark value of Cronbach's *α* value of ≥0.7 is commonly used to indicate that relatively most of the items measure the same construct [23]. In addition, the receiver operating characteristic (ROC) curve analysis of the SPSS software was used to identify and compare the sensitivity and specificity of the questions of the survey [24, 25]. The value of the area under the ROC curve (AUC), calculated through the ROC analysis, is an effective measure that reflects the accuracy of the test where the greater the value, the more reliable the test [26, 27]. The interpretation of the following values of AUC was used in this study: 1.00–0.9 = excellent test of a perfect sensitivity and specificity; 0.89–0.8 = accurate; 0.79–0.7 = not that accurate; 0.69–0.6 = rare to use; and 0.59 or below = worthless test that does not predict outcomes very well [24–28]. Furthermore, the ROC analysis was conducted to determine the total cut-off score where the state of having knowledge about OT can be identified and the range of scores where the level of knowledge can be described as average, poor (below average), and excellent (above average) [28].

## 3. Results

The total number of participants was 829 with a mean age of 32 ± 11.6 yrs (age range 18–80 yrs). The mean total score of participants in the survey was 53.1 ± 10.4, and the total scores of the sample ranged 19–78 out of 80. Numbers, percentages, and mean total scores of participants as arranged by subgroups are displayed in Table 1. The sample included 222 (26.8%) personnel who have background knowledge in healthcare (i.e., physicians, nurses, pharmacists and pharmacologists, rehabilitation specialists, dentists, and paraprofessionals). There were 146 (17.6%) clients who had received or were receiving OT services at the time of conducting the study. Lay people from all other disciplines, and who had never received OT services, constituted 55.6% (*n* = 461) of the sample. Through the subsequent sections, the 40 statements/questions of the main sections 4, 5, and 6 of the questionnaire will be presented along with a focus on presenting the highest percentage of three responses (Yes, No, and I do not know) against each statement/question.

### 3.1. Reliability and Accuracy of the Survey Questions and Cut-Off Score

The results showed that all of the main subscales of the survey were characterized by high values of Cronbach's alpha, which confirmed the reliability of the survey in all of its three main subscales. *Cronbach's α* values were 0.845, 0.831, and 0.765 for sections 4, 5, and 6, respectively.

**Figure 1:**
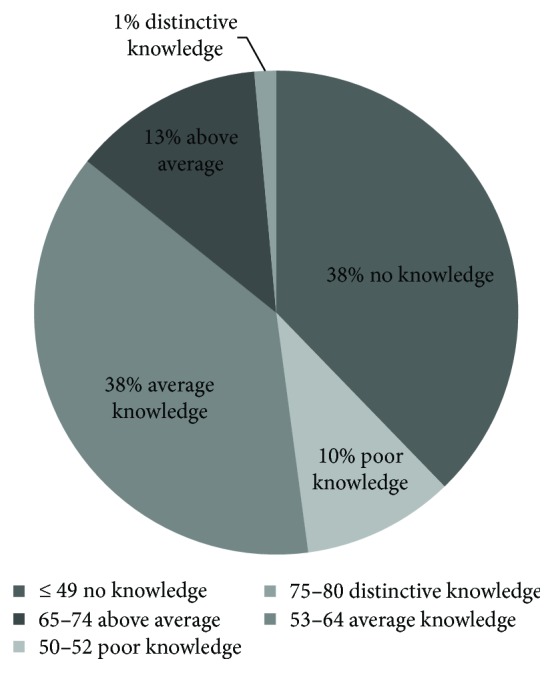
Level of knowledge and awareness of OT.

The AUC of the questions of the survey was AUC = 0.8 which meant that the survey presented an accurate tool in measuring the level of knowledge and awareness about OT. The calculated cut-off score using the ROC analysis was 49.5. Thus, all participants who scored <50 (*n* = 313) were considered not to know or to be aware about OT. The levels of knowledge were determined using the calculated cut-off score of 50 and the overall sample mean and SD scores of 53.1 ± 10.4. The 95% confidence interval was correspondent with the scores ranging 32–74. Accordingly, the categorization of the levels of knowledge was as follows: no knowledge (≤49), poor knowledge (50–52), average knowledge (53–64), above average (65–74), and distinctive knowledge (75–80). As shown in Figure 1, 48% of the sample had poor or no knowledge about OT.

### 3.2. Awareness and Sources of Knowledge about OT

**Figure 2:**
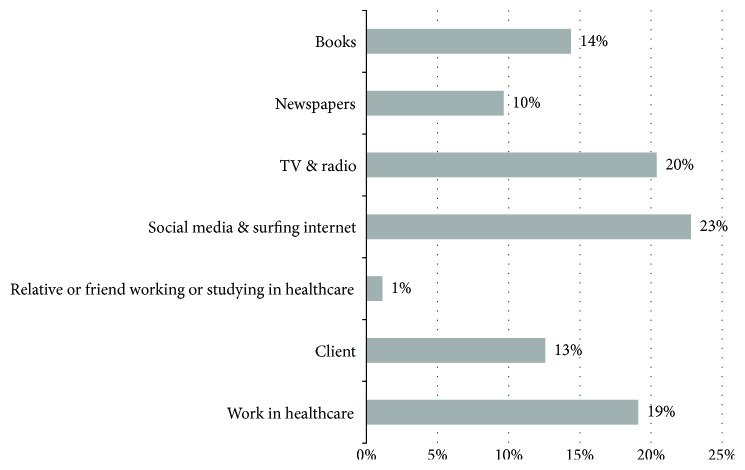
Sources of knowledge and awareness of OT.

Forty-five and a half percent (45.5%) of participants reported not to have any prior knowledge about OT, and 28.3% of participants stated that this was their first time hearing about OT. Sources of knowledge about OT as reported by participants were as follows: working in healthcare, receiving OT services, a relative or a friend working or studying in healthcare, social media and the internet, TV or radio, and books (Figure 2).

### 3.3. Knowledge about Aims and Services Provided in OT

Most participants (63%–68%) knew that OT aims at maximizing the level of participation in meaningful occupations by maintaining and/or developing performance skills that assist clients to adapt to the new health condition and that it is a comprehensive profession that targets social, psychosocial, motor, and mental aspects of health. Participants defined OT as one of the rehabilitation professions that employs a client-centered approach to treatment and that targets all age groups.

The majority of participants (53%) indicated that OTRs work at a preventative level of care and that assessment, measurement, and alteration of wheelchairs and other mobility aids were part of the services provided in OT. The prescription of equipment for the facilitation of performance of daily life activities was perceived to be one of the services provided in OT by 48% of participants while 45% perceived that the organization and running of group therapy sessions were part of OT services.

Most of the participants did not know whether the fabrication of splints was an OT service (48%). The same was true of environmental adaptations, where 50% of participants' answers fell under the category “I do not know.”

### 3.4. Knowledge concerning Fields of OT and Places of Service Provision

Over half (55%) of the sample did not have any knowledge concerning places that provided OT services. Most participants (77%) believed that OT services were mainly provided in hospitals, then community-based rehabilitation (CBR) centers (67%), followed by centers for special education (60%), nursing homes (55%), and mental health institutions (48%). However, only 32% of the participants reported that OT services could be provided in schools.

Most participants reported that OT was a profession that was concerned with pediatric conditions, then teenagers, and lastly older people with percentages of 73%, 70%, and 69%, respectively.

Over half (58% and 53%) of the participants believed that OT was mainly concerned with the treatment of neurological and physical conditions, respectively. However, the majority of participants' answers (44%) were “No” concerning the statement: OT is concerned with the provision of services for mental and psychological health conditions.

### 3.5. Common Misconceptions about OT

**Figure 3:**
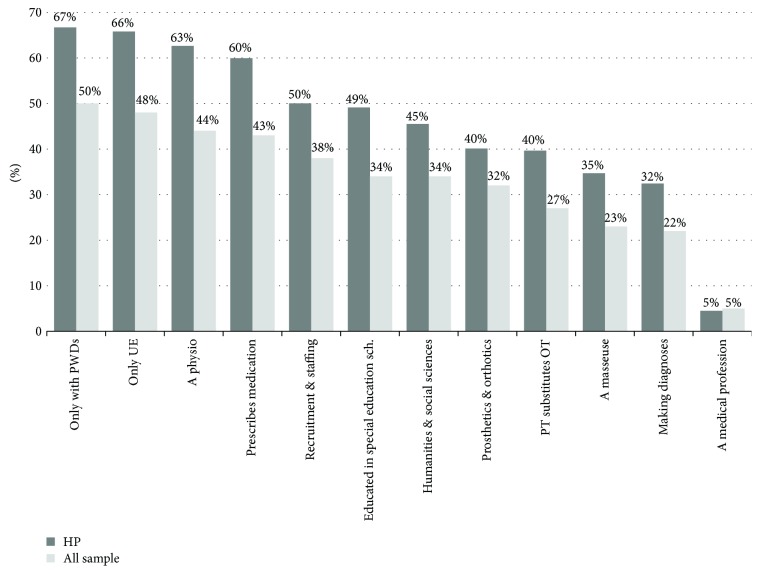
Misconceptions about OT in healthcare personnel subgroup (HP) and the whole sample.

The common misconceptions about OT were that OTRs are solely concerned with people with disabilities (50%), OT is merely focused on the treatment of the conditions of the upper extremity (48%), OTRs are physiotherapists (44%), the prescription of medications is one of the services provided by OTRs (43%), OT is a profession that is concerned with the provision of recruitment and staffing services (38%), an OTR is certified from the school of educational studies (34%), OT is one of the professions related to humanities and social sciences (34%), an OTR is the same as an orthotics and prosthetics specialist (32%), and a physiotherapist can perform/substitute for the role of an OTR (27%) (Figure 3).

Most responses were positive in identifying the following statements as misconceptions about OT which were that OT is a medical profession (78%), an OTR is mainly a masseuse who provides professional massage treatment (47%), and an OTR diagnoses the client's condition prior to provision of services (44%) (Figure 3).

### 3.6. Significance of the Effect of Gender, Age, Educational Level, Area, and Status on the Total Score

The results of the MANOVA revealed significant differences in scores attributed to the effect of independent variables of age, educational level, and status. However, there were no significant differences between subgroup scores as arranged by gender and area (Table 1).

Looking closely at the cross tabulation between the age subgroups and the status subgroups, the majority of participants (*n* = 178, 63%) of the A age subgroup were from the lay subgroup. A similar pattern was revealed for the D age subgroup where the majority were from the lay subgroup (*n* = 61, 65%). The highest percentage of HP participants was concentrated in the B age subgroup (*n* = 98, 44%), and the B age subgroup also contained the largest percentage of clients (*n* = 43, 29%). Thus, the status (clients, lay, and HP) variables were the main factors in causing the significant difference between the mean scores of the age subgroups.

### 3.7. Effect of Background in Healthcare on the Total Score

The demographics of subgroups included in the HP group are displayed in Table 1. Less than a third of the HP group (*n* = 70, 31.5%) reported not to have any prior knowledge about OT, while 34 (15.3%) of them stated that it was the first time for them to hear about OT and thus were not aware of OT. The percentages of misconceptions about OT in the HP subgroup were reflective of the percentages of misconceptions about OT in the whole sample (Figure 3).

**Figure 4:**
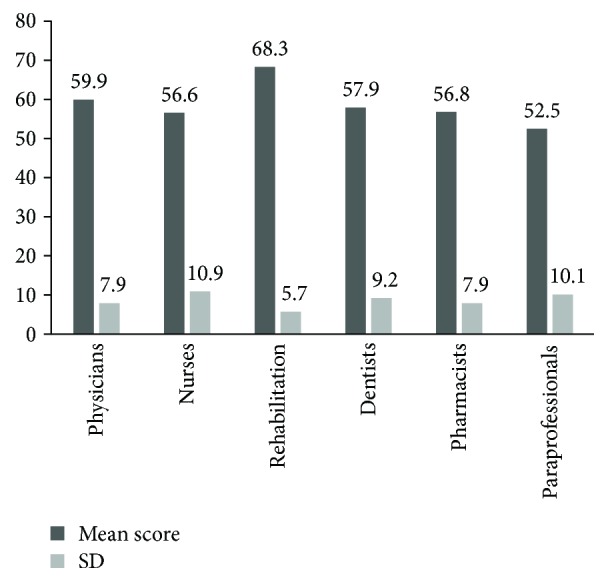
Mean scores and standard deviations of healthcare personnel subgroups.

The MANOVA revealed a significant difference between the scores of subgroups of healthcare backgrounds (*p* ≤ 0.001). The Tukey post hoc test revealed that there were significant differences between the score of the rehab subgroup and all other subgroups where the *p* values were *p* ≤ 0.001. There was also a significant difference between the scores of the physicians and paraprofessionals subgroups (*p* ≤ 0.001), where the mean score of the physicians' subgroup was higher Figure 4.

## 4. Discussion

This study has shown that the role of occupational therapists is still not well recognized in Jordan, which resonates with the results of previous studies [17, 18]. The following subsections will present further interpretations of the sources and implications of this main result in light of the literature.

### 4.1. Role of Professional Bodies in Preserving OT Identity and Prevent Role Confusion

This study found that there was confusion concerning the role of OTRs among the public and even among clients and other healthcare professionals, which resonated with the findings of Abu Tariah et al. [18] and Katz et al. [29]. In Jordan, some special education specialists pursue the role of OTRs and in some healthcare facilities, the role of an OTR is carried out by physiotherapists. Anecdotal evidence from practicing OTRs in Jordan suggests that OTRs may even face several situations where they have to compromise their professional identity to preserve their jobs. For example, in some institutions in Jordan, OTRs have been asked to pursue the role of physiotherapists, special education specialists, or teachers in addition to their duties as OTRs to reduce the amount of paid wages. Thus, the clinical reasoning skills of the OTRs could be directed at implementing practices far from the original philosophy of OT in order to meet the cultural expectations and conceptualization of OT [11–13]. Responses such as this could promote confusion about OT and moves OT practice away from its original underpinnings where a new distorted definition becomes a self-fulfilling prophecy [17, 30]. This, in return, promotes a lack of awareness and knowledge about OT and feeds a cycle that further devalues the OT role [31].

The Ministry of Health (MOH) is the overarching body of issuing certification for all healthcare professionals in Jordan. Currently, the regulation number (84) for governing occupational therapy practice, issued by the MOH [32], does not clearly delineate the role of OTRs nor does support the autonomy of OT practice. The boundaries between practices of healthcare professions need to be delineated [18, 33]. The lack of an active role of OT representative bodies is one of the causes of the lack of public awareness and knowledge about OT [6]. There is a need for legislative standards to be established by professional bodies to define and preserve the OT scope of practice and the rights of OTRs [6, 33].

This study showed that participants' main source of knowledge about OT was the media (social media, TV, and radio) which was also the case in the studies conducted by Olaoye et al. [6] and Patel and Shriber [34] who also found prevailing misconceptions about OT. In Jordan, the Jordanian Society of Occupational Therapy (JSOT) is the relevant body that set plans and implement activities directed at promoting the awareness of the definition and concepts of the OT profession as defined by the World Federation of Occupational Therapists (WFOT). The JSOT may need to consider working on the culturalization of certain concepts. For example, occupational therapy is usually misconceived as aimed at providing jobs because of linguistics ambiguity/confusion associated with the word “occupation” in Arabic. Media panels, social media in particular, could be creatively and intensively used to market and promote an accurate image of OT [18].

### 4.2. Creating Channels of Communication and Collaboration

The knowledge of rehabilitation professionals about OT was the highest among all healthcare subgroups in the current investigation. This result resonated with the results of Abu Tariah et al. [18] who found physiotherapists to have a higher level of knowledge about OT than nurses and physicians. However, it was not expected in this study that the level of knowledge and awareness of other healthcare professional subgroups were similar to the knowledge level of clients. Abu Tariah et al. [18] and AlHeresh and Nikpoulos [17] described the level of knowledge of Jordanian healthcare professionals to be limited. It has been over five years since those studies have been conducted, and it was unexpected to find that the level of knowledge of Jordanian healthcare professionals has not developed much regarding OT. Such a result reflects a lack of communication channels between OTRs and other healthcare professionals [18]. This can restrict interdisciplinary work which would affect the quality and comprehensiveness of services provided for clients [4, 6, 18].

In this study, the value of OT was unacknowledged in school-based settings and this mirrors the results found by Vincent et al. [14] and Benson et al. [15]. Jackman and Stagnitti [4] found that students who needed OT intervention did not receive the required intervention due to the lack of school teachers' knowledge about OT. In contrast, Olaoye et al. [6] also found little knowledge among healthcare undergraduate students but in this study, participants perceived OT services to be exclusively provided in schools and rehabilitation homes. Another finding of this study was that there was a limited awareness of the role of OTRs in mental and psychiatric health settings, in particular. This result resonated with what was found by Fossey [8], who attributed this reduced awareness to the lack of shared education and dialogue opportunities among members of the multidisciplinary team. OTRs have a duty and an obligation to set goals and intervention plans in collaboration with other members of the healthcare team (including family members, clients, teachers, and other healthcare professionals) [2, 15]. This is part of the professional obligations of OT for the delivery of holistic, equal, and client-centered care [2, 3].

Clients in this study were found to have a significantly higher level of knowledge about OT compared to the public though their level of knowledge remained average. This result was unexpected as clients are in direct touch and communicate the most with OTRs who should work with clients using a client-centered approach [3]. Fiss et al. [35] found a discrepancy between the perceptions of the OTRs and those of the clients concerning the focus of treatment. According to Fiss et al. [35], this was attributed to a lack of communication between therapists and clients and an alienation from the approach of client-centeredness. Clients constitute one of the main means of promoting knowledge and awareness about OT, and they can be described as “OT ambassadors” ([33]: p. 17). In their study, Belik et al. [33] found that 70% of the participants knew about OT through receiving direct OT services or through a family member who did. Thus, clients need to be treated as an invaluable source to counteract the public lack of knowledge about OT.

### 4.3. Curricula Reevaluation and Interprofessional Education

The finding that rehabilitation specialists had better knowledge about OT can be explained by the fact that they study in the same school as OTRs and have shared lectures and courses together [18]. In training and even work settings, rehabilitation students and specialists work and train together in the overarching divisions of rehabilitation [18]. Thus, the curricula of other healthcare professions (e.g., medicine, nursing, counseling, and para-professions) need to include courses directed to familiarize students with rehabilitation specialties and interdisciplinary work [2, 5, 17, 18]. Patel and Shriber [34] suggest that direct contact with OTRs can be the best method for promoting knowledge about OT. Rotational clinical placements constitute another interprofessional mechanism of teaching [6]. In addition, promoting awareness about misconceptions about OT that were found in the current investigation, for example, will promote accurate knowledge about OT and its standards among other healthcare professionals [5, 8].

### 4.4. Directing Future Effort and Research to Promote Knowledge about OT

The results of this study suggest that it is the responsibility of OTRs and the JSOT to promote their profession, in order to counteract the lack of knowledge about OT in the public and among members of the multidisciplinary team. This study did not target OTRs' perception and knowledge of OT and how OTRs define OT in Jordan, which would be an essential topic to be targeted in future research.

## Figures and Tables

**Table 1:** Mean scores and SD of subgroups and *p* values.

	Significance (*p* value)	Number & (percentage)	Code	Age *μ* and SD	Score *μ* and SD
Gender	**0.16**				
Males		474 (57.2%)	M	33.2 ± 12.3	52.3 ± 10.4
Females		355 (42.8%)	F	30.4 ± 10.4	54.2 ± 10.4
Age groups	**0.004** ^∗^				
18–24		284 (34.4%)	A	21.6 ± 2	51.5 ± 9.9
25–34		266 (32.1%)	B	28.9 ± 3.1	54.4 ± 10.6
35–44		145 (17.5%)	C	38.7 ± 2.9	53.1 ± 10.4
45–54		94 (11.3%)	D	49.4 ± 3.5	51.9 ± 10.2
55+		40 (4.8%)	E	61.9 ± 6.1	58.1 ± 11.9
Educational level	**0.021** ^∗^				
School (primary & elementary)		56 (6.8%)	Sch	36.6 ± 15.5	50.1 ± 10.2
High school		160 (19.3%)	HS	33.9 ± 13.2	49.6 ± 9.9
Associate degrees		119 (14.4%)	Asso	37.9 ± 11.6	52.4 ± 10.4
Bachelor degrees		459 (55.4%)	Bch	28.8 ± 9.3	54.6 ± 10.2
Master and doctoral degrees		35 (4.2%)	MA+	37.7 ± 9.3	56 ± 11.3
Status	**0.007** ^∗^				
Clients		146 (17.6%)	Client	37.3 ± 13.9	55.5 ± 8.2
Lay people		461 (55.6%)	Lay	31.5 ± 11.4	49.8 ± 9.9
Healthcare background		222 (26.8%)	HP	29.7 ± 9	58.4 ± 10.3
Physicians		*43 (19.4%)*			
Nurses		*46 (20.7%)*			
Rehabilitation specialists		*38 (17.1%)*			
Dentists		*17 (7.7%)*			
Pharmacists & pharmacologists		*23 (10.4%)*			
Paraprofessionals		*55 (24.8%)*			
Area	**0.544**				
Northern		221 (26.7%)	North	31.9 ± 10.9	52 ± 10
Central		436 (52.6%)	Cen	32.5 ± 11.6	54.5 ± 10.6
Southern		93 (11.2%)	South	32.3 ± 13.8	51.1 ± 10.8
Unspecified		79 (9.5%)	Unsp	29.4 ± 10.5	51 ± 10

*Note. n* = number; *μ* = mean; SD = standard deviation. Age groups in years. ^∗^Significant on the 0.05 level.
